# Training robust T1-weighted magnetic resonance imaging liver segmentation models using ensembles of datasets with different contrast protocols and liver disease etiologies

**DOI:** 10.1038/s41598-024-71674-y

**Published:** 2024-09-09

**Authors:** Nihil Patel, Adrian Celaya, Mohamed Eltaher, Rachel Glenn, Kari Brewer Savannah, Kristy K. Brock, Jessica I. Sanchez, Tiffany L. Calderone, Darrel Cleere, Ahmed Elsaiey, Matthew Cagley, Nakul Gupta, David Victor, Laura Beretta, Eugene J. Koay, Tucker J. Netherton, David T. Fuentes

**Affiliations:** 1 Department of Imaging Physics, The University of Texas MD Anderson Cancer Center, Houston, Texas USA; 2 Department of Computational Applied Mathematics and Operations Research, Rice University, Houston, Texas USA; 3 Department of Molecular and Cellular Oncology, The University of Texas MD Anderson Cancer Center, Houston, Texas USA; 4 Department of Gastroenterology, Houston Methodist Hospital, Houston, Texas USA; 5 Department of Radiation Oncology, The University of Texas MD Anderson Cancer Center, Houston, Texas USA; 6 Department of Radiology, Houston Methodist Hospital, Houston, Texas USA; 7 Department of Radiation Physics, The University of Texas MD Anderson Cancer Center, Houston, Texas USA; 8 Department of Molecular and Cellular Oncology, The University of Texas MD Anderson Cancer Center, Houston, Texas USA

**Keywords:** Liver segmentation, T1-weighted MRI, Deep learning, Robustness, Multi-dataset training, Liver model, Computer science, Cancer imaging

## Abstract

Image segmentation of the liver is an important step in treatment planning for liver cancer. However, manual segmentation at a large scale is not practical, leading to increasing reliance on deep learning models to automatically segment the liver. This manuscript develops a generalizable deep learning model to segment the liver on T1-weighted MR images. In particular, three distinct deep learning architectures (nnUNet, PocketNet, Swin UNETR) were considered using data gathered from six geographically different institutions. A total of 819 T1-weighted MR images were gathered from both public and internal sources. Our experiments compared each architecture’s testing performance when trained both intra-institutionally and inter-institutionally. Models trained using nnUNet and its PocketNet variant achieved mean Dice-Sorensen similarity coefficients>0.9 on both intra- and inter-institutional test set data. The performance of these models suggests that nnUNet and PocketNet liver segmentation models trained on a large and diverse collection of T1-weighted MR images would on average achieve good intra-institutional segmentation performance.

## Introduction

The American Cancer Society has reported liver cancer as one of the leading causes of cancer deaths in the U.S., accounting for nearly 30,000 deaths in 2023^1^. Accurate delineation of the liver and tumor is essential for treatment planning^2,3^. Indeed, liver and tumor segmentation methods are crucial in several treatment strategies, such as Y-90 radioembolization^4,5^, radio-frequency ablation^6^, percutaneous ethanol injection^7^, and surgical intervention^8,9^. Accurate liver segmentation is also important for other aspects of treatment like early diagnosis and assessing key indicators like liver fat^10,11^. Although the gold-standard segmentation method is manual delineation by a trained radiologist, this method is time-consuming, less reproducible, and prone to inter and intra-observer variability^12–14^.

In recent years, deep learning models have been trained to perform automated liver segmentation as an alternative to manual delineation. Jansen et al. used a fully convolutional network as part of a liver metastasis detection pipeline to achieve a 0.95 Dice-Sorensen coefficient (DSC) when trained on 55 DCE-MRI series^15^. Isensee et al. submitted a self-configuring nnUNet framework to the LiTS and CHAOS challenges^16^. They finished first in both challenges, scoring mean DSCs of 0.95 on 131 CT series in the LiTS challenge and 0.75 on 60 MRI series in the CHAOS challenge^16–18^. Bibars et al. used the CT images in the LiTS and CHAOS datasets to pretrain the encoder of a 2D U-Net and then fine-tuned the decoder on MRIs from the Duke Liver Dataset (DLDS), achieving a mean DSC of 0.88^17–20^. Lambert et al. trained anisotropic hybrid U-Nets (AHUNets) with 2D encoders and 3D decoders on the ATLAS dataset on the task of segmenting both the liver and the tumor^21,22^, achieving a mean DSC of 0.94, Hausdorff distance of 2.85 mm, and surface DSC of 0.81 on the liver segmentation task. Hossain et al. trained a 2D cascaded network on all 40 T1-weighted MRI series in the CHAOS dataset using five-fold cross-validation and data augmentation. They achieved a mean DSC of 0.95 when segmenting the liver^23^. Due to the relatively small size of publicly available MRI datasets, it is not uncommon for researchers to use more internal institutional data. Kart et al. trained a nnUNet on a dataset of 400 T1-weighted MR images and achieved a mean DSC of 0.98 on a liver subtask of abdominal organ segmentation^24^. Some common limitations in all of these previously mentioned datasets are that they are either obtained from healthy individuals, which limits the ability of trained models to generalize to MRIs from liver cancer patients, or are from a single institution, making models less robust to different imaging sequences and protocols.

Because liver tumors have different etiological factors and morphologies^25^, their effects on the shape, boundaries, and volume of the liver and surrounding structures can vary significantly. Therefore, a model that is robust to these variations must be trained on imaging data from as many unique patients with as many different etiologies as possible. Recently, Wasserthal et al. unveiled TotalSegmentator, a single nnUNet model trained on CT images from 1,204 patients, 655 of which had six different pathologic diagnoses, each with 104 labeled anatomical structures; TotalSegmentator achieved a mean DSC of 0.96 when tested on a liver CT segmentation sub-task of the Beyond the Cranial Vault Challenge^26,27^. While this large, diverse dataset helps mitigate the limitations of the previously mentioned work, it consists of CT images, leaving a need for a similar dataset with MRIs.

This work addresses the previously mentioned limitations by curating a large, multi-institutional, and heterogeneous set of 819 T1-weighted liver MRIs and training robust deep-learning models for automatically segmenting the liver. This dataset comes from various patients and healthy subjects obtained from publicly available and internal (from our institutions) imaging data. Our results show that the variation and diversity in the imaging sequences, artifacts, and contrast agents’ protocols across the dataset allow us to train a robust set of deep learning models for auto-contouring the liver. By analyzing a diverse set of MRI sequences, we aim to improve the reproducibility and consistency of liver segmentation, addressing previous studies’ limitations and enhancing deep learning models’ accuracy and reliability.

## Materials and methods

### Data curation and description

The inclusion criteria for MR images into our dataset are as follows:

1. The entire liver must be visible in the image.
2. All eight liver segments must be present. For example, there is no history of hepatectomy or lobectomy before image acquisition.
3. The image quality must be high enough such that the boundary of the liver is identifiable without using a pre-existing contour.

We manually inspected each image to determine if it met the selection criteria. This process also included using relevant patient and dataset metadata. The primary indicators to identify the liver segments include the presence or absence of the left and right portal veins and tissue homogeneity. We excluded images from patients who had undergone hepatectomy or lobectomy.

This process resulted in a total of 819 T1-weighted MRIs from 312 patients. Of these, 72 patients had cirrhosis, a risk factor and common finding in patients with primary hepatocellular carcinoma, who underwent MRI obtained from the Duke Cancer Institute (data collected from the Duke Liver Dataset [DLDS])^19^. Another 34 patients with liver cancer were obtained from The University of Texas MD Anderson Cancer Center. An additional 71 patients with hepatocellular carcinoma were collected from Houston Methodist Hospital. Fifty-eight anonymized patients from the A Tumor and Liver Automatic Segmentation (ATLAS) dataset with hepatocellular carcinoma were obtained from Bourgogne University in Dijon^22^. Another 57 patients with “abdominal tumors/abnormalities” were obtained from the Longgang District People’s Hospital in China with a protocol approved by the hospital’s Research Ethics Committee (data collected from the Abdominal Multi-Organ Segmentation [AMOS] dataset)^28^. Although a small subset of these patients’ scans showed tumor growth and lesions on the liver itself, most patients had unrelated abnormalities. Finally, 20 healthy individuals were collected from the Dokuz Eylul University Hospital’s Department of Radiology in Izmir, Turkey, using an Institutional Review Board-approved protocol (data collected from the Combined Healthy Abdominal Organ Segmentation [CHAOS] dataset)^18^.

**Fig. 1:**
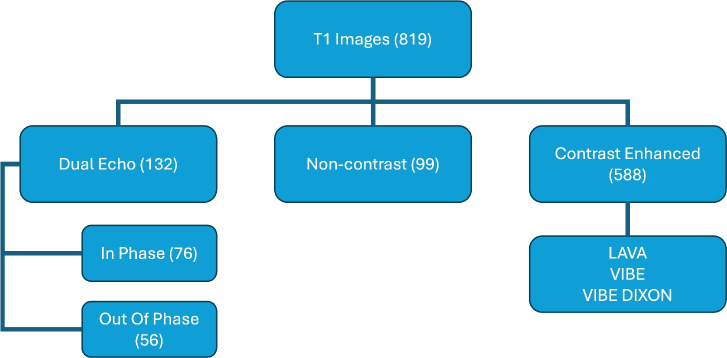
Summary of T1-w MRI sequences used in our study.

Ranges of repetition times, echo times, and contrast agents’ protocol of the public datasets used are provided (whenever available in their corresponding paper) in Table 1. Figure 1 and Table 2 further summarize the datasets we used.

**Table 1:** Summary of echo time (TE), repetition time (TR), and contrast agents used in MRIs.

Dataset		TR (ms)	TE (ms)	Contrast agent	Acquisition timing
CHAOS		–	–	–	–
DLDS	In phase	3.84 – 175	2.46 – 7.38	Gadobenate dimeglumine (0.1 mL/kg) Gadoxetate disodium (0.05 mL/kg) Rate of infusion 2 mL/s	Arterial phase at 15 seconds; portal venous phase at 70 seconds
	Out of phase	3.84 – 175	1.23 – 6.15		
	Non-contrast	3.46 – 9.20	1.07 – 3.13		
	Contrast-enhanced	2.83 – 6.96	1.23 – 3.27		
AMOS		–	–	–	–
ATLAS		3.09 – 6.78	1.07 – 4.19	Gadolinium-based contrast	Arterial (early, late) at 12-30 seconds; portal venous phase at 65–70 seconds; delayed at 180-300 seconds
Houston Methodist		2.79 – 6.05	1.23 – 3.12	Gadolinium-based contrast	Arterial phase obtained with bolus tracking and triggered when contrast detected in abdominal aorta; portal venous phase at 30 seconds after aterial phase; delayed at 300-600 seconds
MD Anderson		2.65 – 4.69	1.02 – 2.41	Gadolinium-based contrast	Arterial at 30 seconds; portal venous phase at 60 seconds; delayed at 180 seconds

**Table 2:** T1-weighted dataset breakdown and distribution.

Dataset	No. patients	No. images	Participants’ findings	Voxel spacing range, mm			No. duplicates	Cause of duplication	Image distribution
				*x*	*y*	*z*			
CHAOS	20	40	Healthy individuals	0.7 – 0.8	0.7 – 0.8	0.7 – 0.8	20	Dual-phase images (each phase = 1 image)	In phase: $n=20$; out of phase: $n=20$
DLDS	72	210	Cirrhosis	0.6 – 1.8	0.6 – 1.8	2.4 – 10.0	64	Different types of contrast	In phase non-fat saturation: $n=56$; late dynamic: $n=2$; out of phase: $n=36$; pre-contrast fat suppressed: $n=54$; early arterial: $n=1$; mid-arterial: $n=3$; portal venous: $n=58$
AMOS	57	57	Liver tumor (small sample)	0.6 – 2.0	0.6 – 3.0	0.8 – 3.0	0	–	Not provided
ATLAS	58	58	Hepatocellular carcinoma	0.6	0.6	1.4	0	–	Fat saturated: $n=58$ (pre-contrast, arterial, portal venous)
Houston Methodist	71	352	Hepatocellular carcinoma	0.6 – 1.4	0.6 – 1.4	2.2 – 4	70	Different scanning protocols	Delayed post-contrast fat suppressed: $n=352$
MD Anderson	34	102	Liver tumor	0.6 – 1.6	0.6 – 1.6	2.0 – 3.5	34	Different phases of contrast	Pre-contrast: $n=34$; arterial phase: $n=34$; portal venous phase: $n=34$

### Network architectures

Our experiments involved training three different liver segmentation models: Swin UNETR, nnUNet, and PocketNet.

#### Swin UNETR

The Swin UNETR model is a deep learning architecture designed for medical image segmentation tasks, integrating the Swin Transformer with the UNETR framework^29,30^. It leverages the Swin Transformer’s hierarchical feature representation and shift windowing mechanisms to capture global context and local details within medical images effectively. The model’s architecture combines the strengths of vision transformers in encoding long-range dependencies and the U-Net’s efficient up-sampling and localization capabilities, resulting in improved medical imaging segmentation.

#### nnUNet

Since its introduction, nnUNet has become a popular tool for use in medical image segmentation because its ability to automatically configure a preprocessing and deep learning training pipeline based on the properties of its training data eases the burden of manually developing models to suit a particular data modality^16^. We chose specifically to train 3D full-resolution U-Nets using nnUNet as a baseline for comparison against the other two models.

#### PocketNet

The PocketNet paradigm was originally proposed to reduce the number of parameters in CNN architectures while maintaining their accuracy^31^. This approach uses the similarity between geometric multigrid methods for solving linear systems arising from discretizing partial differential equations and CNNs to justify keeping the number of features at each resolution constant. In contrast, traditional CNNs double the number of features when going from higher to lower resolutions. As a result, PocketNet architectures reduce the number of parameters in CNN architectures by several orders of magnitude and have been shown to achieve similar accuracy to traditional CNNs. Here, we apply the PocketNet paradigm to the nnUNet architecture and refer to this architecture as PocketNet for the sake of conciseness.

### Preprocessing protocols

We apply the same preprocessing steps for all models and datasets. Namely, we apply the rule-based analysis and preprocessing steps proposed by the nnUNet architecture authors. This resulting target spacing and patch size for each individual and combined dataset are given in Table 3. Because of the increased computational cost of the Swin UNETR architecture vs its CNN counterparts, we use a patch size of 128 $\times$ 128 $\times$ 64.

**Table 3:** Resulting target spacing and patch sizes from applying the rule-based analysis and preprocessing steps proposed by the nnUNet architecture authors.

Dataset	Target spacing (mm)	Patch size (nnUNet and PocketNet only)
AMOS	1.1875 $\times$ 1.1875 $\times$ 3.0	256 $\times$ 128 $\times$ 64
ATLAS	1.0417 $\times$ 1.0417 $\times$ 3.0	256 $\times$ 256 $\times$ 64
CHAOS	1.6992 $\times$ 1.6992 $\times$ 5.5	128 $\times$ 128 $\times$ 32
DLDS	0.7813 $\times$ 0.7813 $\times$ 4.0	256 $\times$ 256 $\times$ 64
MD Anderson	0.7031 $\times$ 0.7031 $\times$ 2.0	256 $\times$ 256 $\times$ 64
Methodist	0.7813 $\times$ 0.7813 $\times$ 2.4	256 $\times$ 256 $\times$ 64
All (Experiment 2)	0.8203 $\times$ 0.8203 $\times$ 2.4	256 $\times$ 256 $\times$ 64

### Hyperparameters, training, and evaluation protocols

We train each model using at least two A100 Nvidia GPUs with a batch size of twice the number of GPUs. All models are trained for at least 1000 epochs and use the same optimization parameters as the nnUNet framework. Apart from the Swin UNETR model, we use deep supervision. Additionally, automatic mixed precision was used during training to reduce the time and memory requirements. All models use the Dice with cross-entropy loss. We use test-time augmentation (average prediction after flipping along each axis) and postprocess the final predictions by taking the largest connected component. To evaluate the validity of each predicted segmentation mask, we use the following metrics: the DSC, 95th percentile Hausdorff distance (HD 95), and surface dice with a tolerance of 2mm. We chose surface DSC specifically to offset the skew that the large internal volume of the liver can have on the DSC^32^.

### Experimental design

Using the data and models described in the prior sections, we perform two experiments to evaluate each model’s performance on MRI liver segmentation when trained on a single dataset and on ensembles of datasets.

#### Experiment 1: single source five-fold cross-validation

In this experiment, we perform a five-fold cross-validation with each model on each dataset separately. For each dataset, we set aside the first 20% of the data as an independent test set, take 10% of the remaining data as a validation set, and train on the remaining image-label pairs. We continue this process until we have test-time predictions for each image in a given dataset.

This experiment aims to determine how each model performs on test images that come from the same distribution as the training data, which will serve as a baseline to compare how the same architectures perform on out-of-distribution examples in the following experiment.

#### Experiment 2: leave-one-dataset-out cross-validation

Following our first experiment, we trained and validated six models on all curated T1-weighted MR images, with each dataset withheld for testing.

While our first experiment would demonstrate how each model would perform when tested on in-distribution samples, our second experiment aims to evaluate our models’ performance when tested on out-of-distribution examples. Our hypothesis with this second experiment is that the test-time performance on the withheld dataset would match or exceed the corresponding results from Experiment 1 only if the images in the training set are of similar quality or contrast protocol type to those of the withheld dataset.

## Results

### Experiment 1: Single source five-fold cross-validation

Table 4 shows each metric’s mean and standard deviation for each model resulting from a five-fold cross-validation on each dataset. We see that the PocketNet and nnUNet architectures generally achieve similar accuracy. However, both of these models outperform the Swin UNETR architecture.

**Fig. 2:**
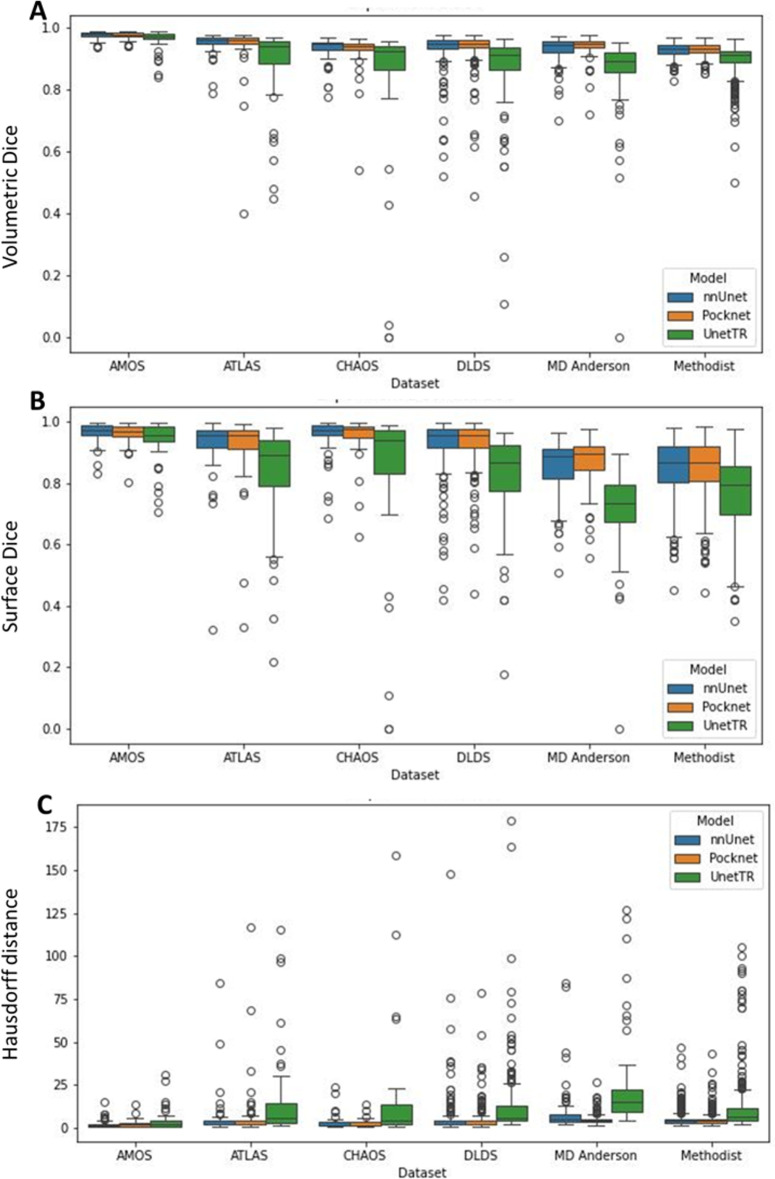
Boxplots for Experiment 1. (**A**) DSC for the six datasets and three models, (**B**) surface DSC for the same, and (**C**) HD 95. We see here that the nnUNet and PocketNet models show comparatively similar variations in accuracy, while the Swin UNETR shows the most variation.

**Fig. 3:**
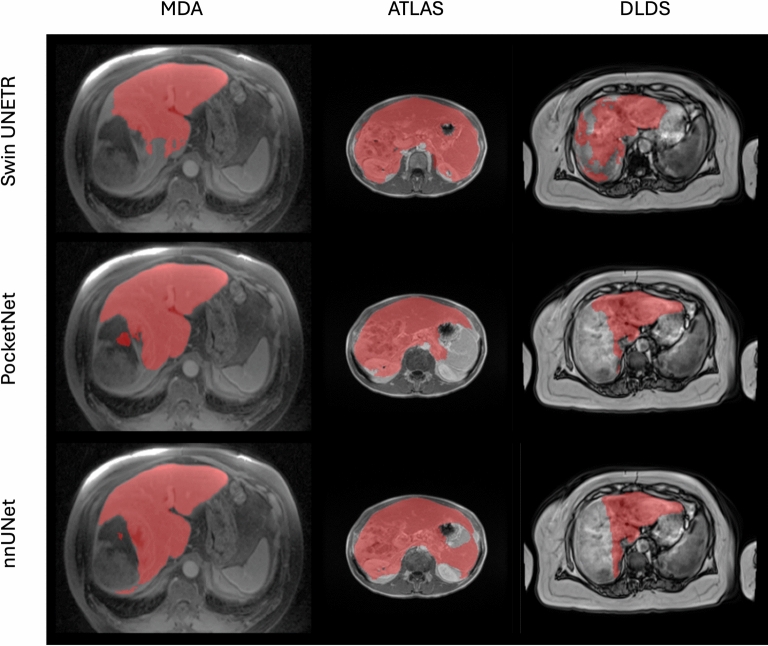
Examples of poorly predicted segmentation masks from all three models in Experiment 1. In the case of the MDA image, we see a large solid lesion on the liver boundary whose signal intensity is close to its surroundings, resulting in under-segmentation. For the ATLAS case, we see close signal intensity between the liver and the spleen, resulting in over-segmentation. For the DLDS case, we see a motion artifact resulting in under-segmentation.

For comparison, Fig. 2 provides boxplots of the DSC, HD 95, and surface DSC for Experiment 1. We see here that the nnUNet and PocketNet models show comparatively similar variations in accuracy, while the Swin UNETR shows the most variation. Outliers were caused primarily by under-segmentation of the liver, especially in the presence of motion or noise artifacts and large complex (solid/ cystic) liver masses, under-segmentation of a tumor or lesion (relatively large lesion along the boundary of the right margin of the liver with signal hypointensity), and over-segmentation of either the abdominal wall or surrounding organs, such as the spleen and kidney. Figure 3 shows the resulting image segmentation quality for a subset of images with these characteristics.

In Fig. 3, all three models performed poorly on the same MR image from the ATLAS dataset, which showed severe over-segmentation of the spleen and other surrounding structures. This common failure is believed to be due to the close similarity of signal intensity between the liver and the spleen and the lack of a distinct boundary between the two organs in this MR image. In the DLDS column of Fig. 3, all three models under-segmented this case, although the Swin UNETR model contoured more of the liver than the other models. In this DLDS case, the imaging shows complex cystic solid masses. In the MDA column, all models under-segmented the right lobe of the liver on a portal venous phase MR image from a patient with a large homogeneous mass occupying this lobe. The Swin UNETR model completely under-segmented the entire liver on an arterial phase MR image from this same patient.

**Fig. 4:**
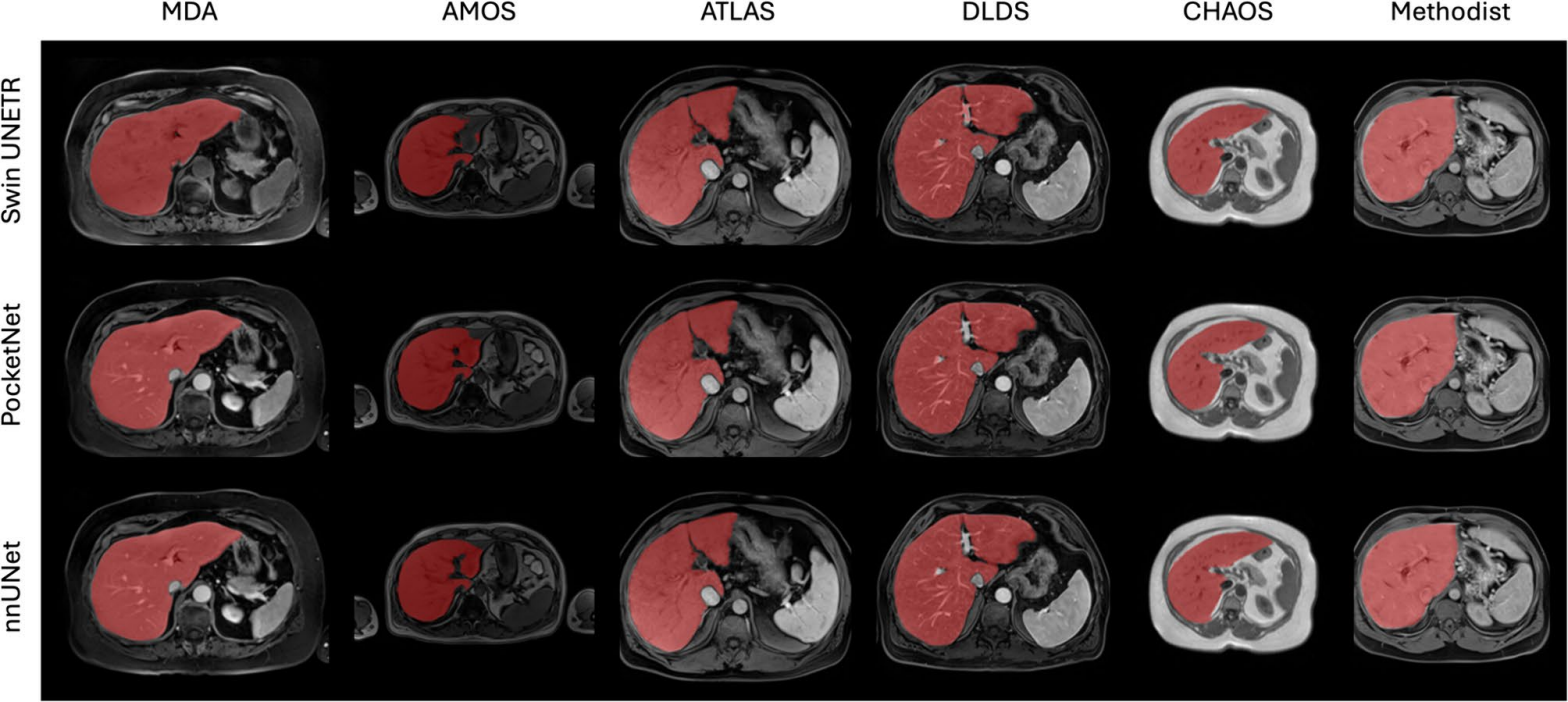
Examples of accurately predicted segmentation masks from Experiment 1.

Figure 4 shows accurate predictions from each model. Notable errors were under-segmentation and over-segmentation of the inferior vena cava, although this discrepancy could be attributed to inter-observer variability across datasets.

**Table 4:** The mean (standard deviation) for each model’s DSC, HD 95, and surface DSC for Experiment 1 - a five-fold cross-validation on each dataset.

Model	Dataset	DSC	HD 95 (mm)	Surface DSC
Swin UNETR	AMOS	0.9634 (0.0304)	4.18 (5.72)	0.9412 (0.0628)
	ATLAS	0.8894 (0.1195)	15.3 (23.9)	0.8326 (0.1591)
	CHAOS	0.8225 (0.2529)	15.9 (31.3)	0.8276 (0.2616)
	DLDS	0.8824 (0.0997)	13.2 (21.1)	0.8337 (0.1191)
	MD Anderson	0.8655 (0.1143)	21.5 (22.2)	0.7210 (0.1218)
	Methodist	0.8969 (0.0491)	11.8 (15.7)	0.7710 (0.1161)
PocketNet	AMOS	0.9738 (0.0119)	2.44 (2.17)	0.9627 (0.0327)
	ATLAS	0.9420 (0.0799)	7.50 (17.6)	0.9221 (0.1109)
	CHAOS	0.9223 (0.0702)	**2.71 (2.57)**	**0.9517 (0.0731)**
	DLDS	**0.9355 (0.0572)**	**4.87 (8.23)**	**0.9343 (0.0731)**
	MD Anderson	**0.9395 (0.0329)**	**5.31 (3.76)**	**0.8755 (0.0747)**
	Methodist	**0.9289 (0.0197)**	**4.92 (4.08)**	**0.8537 (0.0925)**
nnUNet	AMOS	**0.9745 (0.0125)**	**2.42 (2.42)**	**0.9654 (0.0323)**
	ATLAS	**0.9511 (0.0327)**	**5.79 (12.5)**	**0.9268 (0.0986)**
	CHAOS	**0.9278 (0.0440)**	3.51 (4.79)	0.9478 (0.0720)
	DLDS	0.9331 (0.0595)	5.62 (13.0)	0.9229 (0.0854)
	MD Anderson	0.9330 (0.0400)	8.34 (12.4)	0.8528 (0.0895)
	Methodist	0.9279 (0.0205)	5.17 (4.73)	0.8528 (0.0923)

We highlight the best values across all metrics in bold. The PocketNet and nnUNet architectures are comparable and outperform the Swin UNETR model.

### Experiment 2: leave-one-dataset-out cross-validation

Table 5 shows each metric’s mean and standard deviation for each model resulting from a five-fold cross-validation on each dataset. Like with Experiment 1, the PocketNet and nnUNet architectures generally achieve similar accuracy while outperforming the Swin UNETR model.

Recall that our hypothesis for Experiment 2 was that each model’s performance, when tested on a withheld dataset, would match or exceed the corresponding results from Experiment 1 only if the images in the training set were of similar quality or contrast protocol type to those of the withheld dataset. In other words, because of the differences between each dataset, we would expect to see a decrease in accuracy between each model in Experiment 2 vs. 1. This generally appears to be the case for PocketNet and nnUNet, with PocketNet recording overall better accuracy on the CHAOS dataset and nnUNet with the MD Anderson dataset. The Swin UNETR model does not appear to conform to our hypothesis. In this case, Swin UNETR reports improved mean DSC for the ATLAS, CHAOS, and MD Anderson datasets and HD 95 distances for all but the AMOS and MD Anderson datasets.

Figure 6 shows predicted segmentation masks whose DSC is lower than 0.8. We exclude AMOS and ATLAS since all three models achieved a DSC of at least 0.8 for nearly every example. When tested on a low-accuracy case from DLDS, the Swin UNETR model completely undersegmented the target organ, only labeling a tiny sliver of the right liver lobe. PocketNet and nnUNet over-segmented the abdominal region surrounding the front right liver lobe in the same image. We hypothesize that the models performed poorly on this DLDS case due to massive ascites (fluid around the liver) and shrunken cirrhotic liver. In the case of the MD Anderson column in Fig. 6, PocketNet and nnUNet only segmented the right liver lobe. Coincidentally, nnUNet’s outlier was the same MR image that was its outlier when trained on this cohort in Experiment 1. Finally, all three models over-segmented the spleen when tested on their worst case from the Methodist dataset.

Figure 7 shows examples of accurately predicted segmentation masks from withheld images for each model. The most noticeable discrepancies include over-segmentation around the common hepatic duct, over-segmentation of the middle hepatic vein in the CHAOS dataset, and under-segmentation of the left portal vein.

**Table 5:** The mean (standard deviation) for each model’s DSC, HD 95, and surface DSC for Experiment 2 - a leave-one-dataset-out cross-validation.

Model	Dataset	DSC	HD 95 (mm)	Surface DSC
Swin UNETR	AMOS	0.9392 (0.0389)	8.17 (8.30)	0.8948 (0.0760)
	ATLAS	0.9355 (0.0469)	8.35 (12.5)	0.8869 (0.0101)
	CHAOS	0.9008 (0.0533)	6.08 (7.50)	0.9117 (0.0887)
	DLDS	0.8230 (0.1902)	19.9 (24.2)	0.7489 (0.2034)
	MD Anderson	0.8900 (0.0922)	13.4 (14.2)	0.7852 (0.1228)
	Methodist	0.8751 (0.0639)	17.4 (22.0)	0.7355 (0.1222)
PocketNet	AMOS	0.9525 (0.0154)	**4.77 (4.12)**	0.9175 (0.0932)
	ATLAS	0.9471 (0.0279)	5.38 (6.90)	0.9117 (0.0887)
	CHAOS	**0.9328 (0.0314)**	**2.27 (1.34)**	**0.9590 (0.0395)**
	DLDS	**0.9088 (0.1094)**	10.3 (22.5)	**0.8786 (0.1299)**
	MD Anderson	0.9277 (0.0428)	6.90 (6.57)	0.8523 (0.0997)
	Methodist	**0.9083 (0.0312)**	**8.19 (11.8)**	**0.8041 (0.0988)**
nnUNet	AMOS	**0.9572 (0.0144)**	5.26 (6.26)	**0.9347 (0.0527)**
	ATLAS	**0.9557 (0.0142)**	**3.74 (3.42)**	**0.9366 (0.0722)**
	CHAOS	0.9317 (0.0318)	2.44 (1.76)	0.9565 (0.0435)
	DLDS	0.9003 (0.1219)	**9.65 (16.5)**	0.8669 (0.1370)
	MD Anderson	**0.9333 (0.0325)**	**5.77 (4.55)**	**0.8594 (0.0975)**
	Methodist	0.9060 (0.0577)	8.36 (14.3)	0.8030 (0.1074)

We highlight the best values across all metrics in bold. The PocketNet and nnUNet architectures are comparable and outperform the Swin UNETR model.

**Fig. 5:**
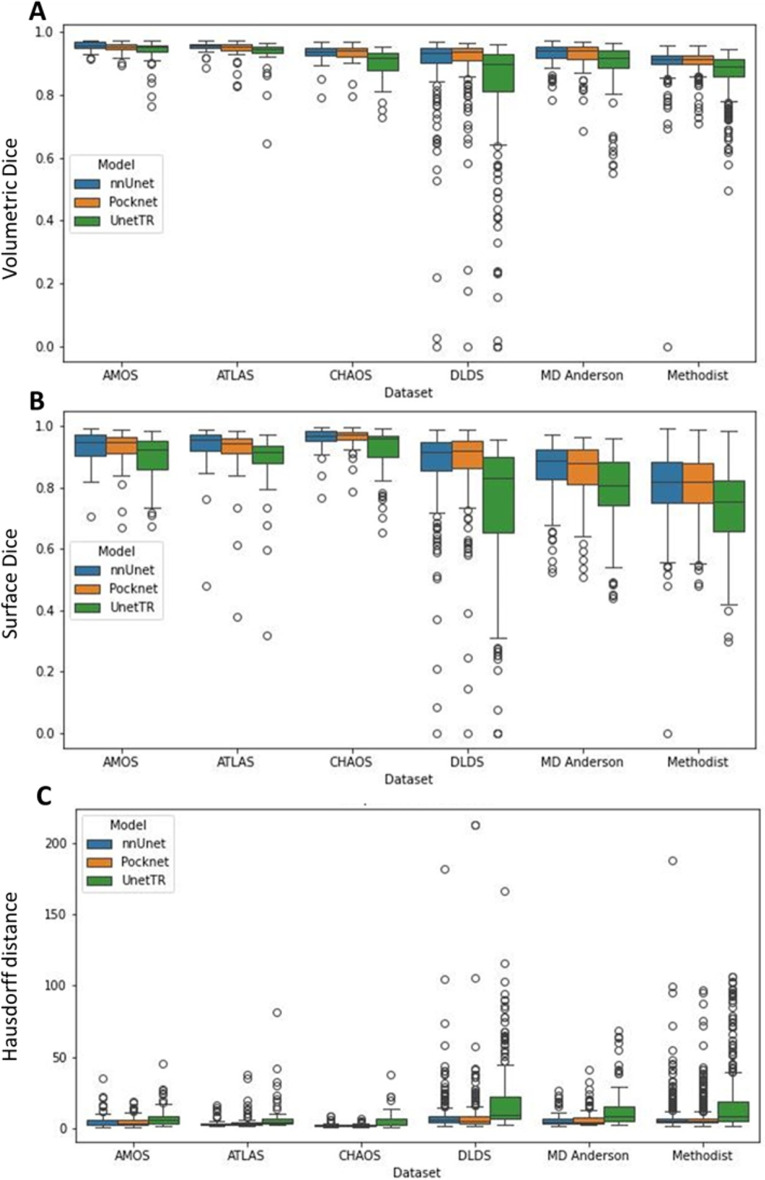
Boxplots for Experiment 2. (**A**) DSC for the six datasets and three models, (**B**) surface DSC for the same, and (**C**) HD 95. We see here that the nnUNet and PocketNet models show comparatively similar variations in accuracy, while the Swin UNETR shows the most variation.

**Fig. 6:**
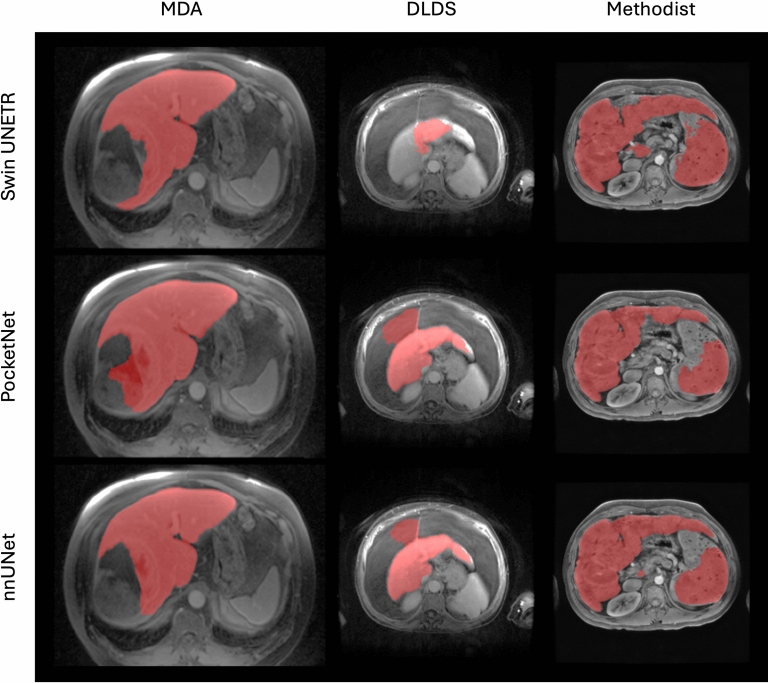
Examples of poorly predicted segmentation masks from all three models in Experiment 2. Like with Experiment 1, we see a large lesion on the liver boundary whose signal intensity is close to its surroundings, resulting in under-segmentation in the same MDA case. In the DLDS case, we see massive ascites (fluid around the liver) and shrunken cirrhotic liver, resulting in under-segmentation for the Swin UNETR model and over-segmentation for the PocketNet and nnUNet models. For the Methodist case, the liver and spleen have similar signal intensities, resulting in over-segmentation.

**Fig. 7:**
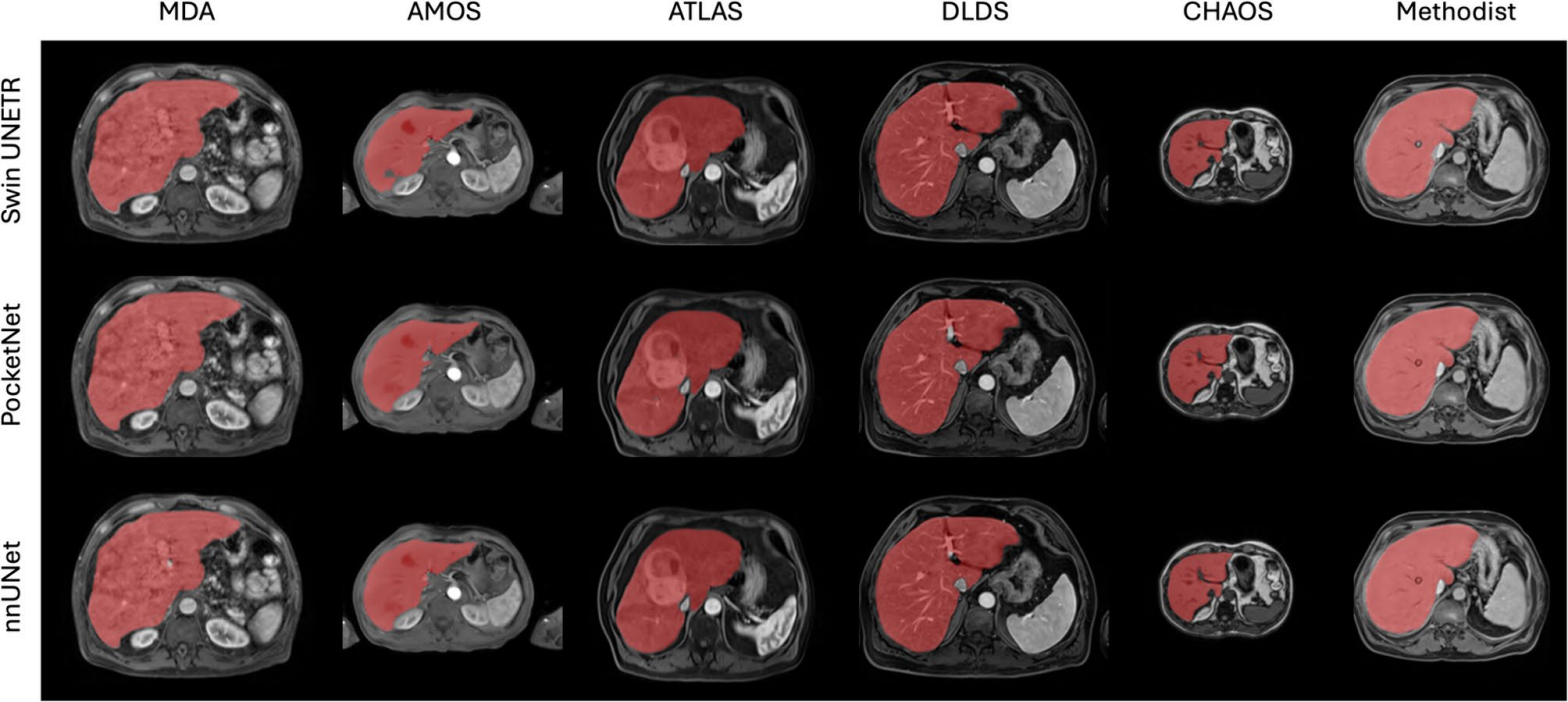
Examples of accurately predicted segmentation masks from Experiment 2.

### Error analysis

Low dice scores (DSC<0.8), indicated in Figs. 2 and 5, were manually reviewed across all models and experiments to characterize failure modes. In this analysis, we found that the most common failure modes are

1. Motion artifacts (Fig. 3 [DLDS]).
2. Massive ascites (fluid around the liver) and shrunken cirrhotic liver (Fig. 6 [DLDS]).
3. Similar signal intensities between the liver and surrounding regions (Fig. 3 [ATLAS]).
4. The presence of a large infiltrative lesion (Figs. 3 and 6 [MDA]).
5. The presence of a hernia.

Table 6 shows the frequency of each failure mode within our dataset. Here, unique image series are considered, i.e. repeat poor performance across models and experiments was counted once. Note that there were seven cases where we did not see any odd pathologies and could not determine why our models produced less accurate liver segmentation masks.

**Table 6:** Characterization of image features that result in low DSC for all three models and both experiments.

Finding	No. images
Motion artifact	9
Ascites and cirrhosis	8
Similar signal intensity	5
Large infiltrative lesion	4
Hernia	2
No odd pathology	7
Total	35

## Discussion

We aimed to use three deep learning architectures and as many T1-weighted MR images as we could gather from multiple institutions to train a robust, accurate liver segmentation model for multiple MRI vendors and liver disease etiologies. From a supervised learning perspective, such a model must be trained on a sufficiently large and diverse cohort of MR images encompassing as many etiologies, contrast agent types, and artifacts as possible. Our Experiment 2 models and their results, when tested on their respective withheld datasets, provided us with an approximation of how each of the three architectures might perform when confronted with a new dataset. Additionally, our results show that PocketNet and nnUNet are effective architectures for training accurate and robust models for MRI liver segmentation. These architectures achieved similar accuracy in Experiments 1 and 2 and showed similar variance.

We hypothesized that the models in Experiment 2 would not outperform those in Experiment 1 because the withheld dataset is sufficiently different from the rest of the training data in each fold of Experiment 2. Our results for Experiment 2 using nnUNet and PocketNet support this hypothesis. We generally see a drop in accuracy for each model (PocketNet and nnUNet) except for the CHAOS dataset with PocketNet and the MD Anderson dataset with nnUNet. However, even in those non-conforming cases, the increase in performance is slight, with the only exception being the HD 95 with nnUNet.

Our hypothesis regarding the accuracy differences in Experiments 1 and 2 using the Swin UNETR model does not hold up as well as with the PocketNet and nnUNet models. One possible explanation is that transformer networks like Swin UNETR are data-hungry^33,34^. The bigger training set size for each fold in Experiment 2 may have helped alleviate this commonly seen challenge with vision transformers. Indeed, the difference in accuracy in the small CHAOS dataset ($n=40$) supports this. In Experiment 1, the Swin UNETR model could only use 32 images for training. On the other hand, this same model had 779 images to train with during Experiment 2.

We might consider the drop in performance observed across all three models when tested on the DLDS in Experiment 2 compared with how they performed when trained only on this dataset in Experiment 1 as supporting evidence for our hypothesis, given the large amounts of motion and susceptibility artifacts that are present in the dataset^19^, more so than any other dataset that we used. These artifacts most probably contributed to the Swin UNETR model’s drop in performance, as its surface DSC values were the lowest of all three models when tested on DLDS in Experiment 2, and these artifacts also likely worsened the performance of PocketNet and nnUNet. However, another reason for the worsened performance of the models could be the liver shape and appearance changes caused by cirrhosis, which would suggest that liver disease etiology was a more significant confounding factor than image quality or contrast type.

Our results present evidence for and against our Experiment 2 hypothesis, and the lack of information regarding contrast types for the AMOS dataset or echo and repetition times for both AMOS and CHAOS are further complications that prevent us from making a proper conclusion on this hypothesis.

Maximum segmentation accuracy is necessary for precise localization and characterization of the liver tissue and the accompanying pathology, which aids radiologists and surgeons in optimizing the diagnosis and staging of the disease, and this is considered the cornerstone of management and treatment planning in terms of surgery and radiological intervention. An automated, robust segmentation model will give a more reproducible estimate of the volumetric measures and extent of liver tissue/lesion than manual or semi-automated methods, as these could be biased or subject to interobserver variability^12–14^. Automated models are still in their developmental stages, and their underperformance regarding segmentation accuracy can result in suboptimal patient outcomes. For example, under-segmentation can lead to the persistence of residual tissue after resection or chemoembolization, whereas over-segmentation can result in unnecessary interventions and inaccurate estimation of the residual liver volume and function during surgical planning^8^. Future work will further evaluate the impact of the observed failure modes in Table 6 on segmentation accuracy.

Of the three architectures tested, our results indicate that PocketNet and nnUNet are effective architectures for training accurate and robust models for MRI liver segmentation. While the Swin UNETR model was not as accurate as its CNN counterparts in either experiment, improving its performance with pre-training on large, publicly available CT datasets like LiTS or TotalSegmentor may be possible. This line of inquiry is a possible direction for future work. The differences in the number of parameters in each architecture are also worth noting. PocketNet has roughly 800,000 parameters, nnUNet has roughly 31,000,000, and Swin UNETR has roughly 62,000,000 parameters. Our results show similar performance between PocketNet (a pocket version of nnUNet) and the full-sized nnUNet, further validating the results from the original PocketNet paper^31^. While PocketNet and nnUNet show similar accuracy, it is also important to point out that the reduced computational cost of PocketNet (from having fewer parameters) makes training and deploying such a model more suitable for resource-constrained environments that might not have access to the latest GPUs (or GPUs in general).

Our work built upon existing research by training the proven nnUNet and its Pocketnet variant on the task of segmenting the liver using 819 T1-weighted MR images gathered mostly from liver cancer patients with different contrast protocols, with performance ranging from comparable to superior when compared against existing models^15,16,20,21,23,24^. However, unlike Lambert et al.’s AHUNets^21^, we did not distinguish between the liver and the tumor and counted the latter as part of the former.

Of the six datasets we used in our experiments, only AMOS, ATLAS, CHAOS, and DLDS are publicly available. As a result, only the results from Experiment 1 with these specific datasets will be reproducible. Furthermore, although curating multiple datasets allowed us to build a sizable and diverse group of MR images for our work, these datasets were labeled by different people. This interobserver variability between ground truth masks is another important confounding factor. Unfortunately, unless one or more trained radiologists are willing to manually edit over 800 liver contours to ensure uniformity across datasets, this limitation has no easy fix.

Work by Isensee et al. that compared the rankings of models submitted to a kidney and kidney tumor segmentation challenge indicated that changes to external parameters such as the learning rate, patch sizes, loss functions, and preprocessing schemes had a more significant impact on performance than changes to actual network architecture^16^. Future work might involve refinement of the “method configuration,” as Isensee et al. collectively referred to these parameters, to determine their effect on liver segmentation accuracy. Additional avenues of exploration include further training of our models on any additional T1-weighted liver MRI datasets that have been made public since the start of our research (i.e., TotalSegmentor MRI^35^), applying our methodology to T2-weighted MRI datasets, training on a combined T1 and T2-weighted dataset, or further cross-sequence fusion across additional imaging modalities. Additionally, future work will also involve image denoising. Cui et al. recently used a 2D CNN and k-space analysis to reduce and remove motion artifacts from corrupted T2-weighted brain MR images^36^. Given both the prevalence of motion artifacts in DLDS and the fact that such artifacts are not uncommon in a clinical setting^19^, an algorithm that can be applied to remove motion artifacts from liver MR images would expedite the training of robust deep learning segmentation models to assist in preventive surgery.

## Conclusions

We sought to train a robust and generalizable liver T1-weighted MRI segmentation model across different contrast protocols and disease etiologies. Of the architectures we trained using an ensemble of curated data drawn from multiple datasets, we found that models trained using PocketNet and nnUNet were the most robust to changes in image and target organ appearance due to a difference in imaging or health factors. We observed this trend across all six datasets, suggesting that any PocketNet or nnUNet model trained on an ensemble of T1-weighted MR images of similar or greater size and diversity will also demonstrate this generalizability.

## Data Availability

The AMOS, ATLAS, CHAOS, and DLDS datasets are publicly available^18,19,22,28^ and can be downloaded using the following links: 1. AMOS: https://amos22.grand-challenge.org/ 2. ATLAS: https://atlas-challenge.u-bourgogne.fr/ 3. CHAOS: https://chaos.grand-challenge.org/ 4. DLDS: https://zenodo.org/records/7774566 The MR images from MD Anderson and Houston Methodist Hospital are not publicly available.

## Code availability

The analysis, preprocessing, training, and postprocessing pipelines and all three models were implemented in Python using the Medical Imaging Segmentation Toolkit (MIST)^31,37,38^. This open-source package is available at https://github.com/mist-medical/MIST or https://pypi.org/project/mist-medical/.
